# High-efficiency photocatalytic CO_2_ reduction in organic–aqueous system: a new insight into the role of water†

**DOI:** 10.1039/c7ra12801k

**Published:** 2018-01-19

**Authors:** Jinliang Lin, Rongying Liao, Junli Xu

**Affiliations:** Department of Chemical and Engineering, Zunyi Normal College 563000 Zunyi P. R. China; Research Institute of Photocatalysis, State Key Laboratory of Photocatalysis, Fuzhou University Fuzhou 350002 P. R. China jinliang_lin@163.com +86-851-28927159 +86-851-28927159

## Abstract

We have first identified a new promotional mechanism of water in the photocatalytic conversion of CO_2_ into CO, which is different from the traditional role of proton source. High efficiency (44.5 μmol h^−1^) achieved through construction of a binary liquid system was determined by systematic research.

## Abstract 2

A high efficiency photocatalytic conversion of CO_2_ into CO has been achieved by construction of a binary liquid system.

## Graphical abstract

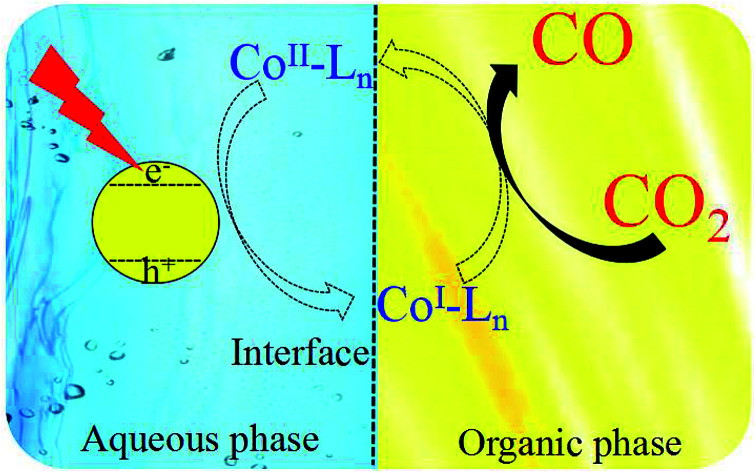

Global warming caused by the increasing CO_2_ concentration in the atmosphere has attracted considerable attention all over the world.^1^ A previous study has demonstrated that CO_2_ could be photocatalytically reduced, mostly in the liquid phase, which is one of the most promising pathways to solve the problems of both environmental pollution and fossil fuel shortage.^2^ Most of the research on CO_2_ photoreduction was related to water as reaction medium or/and aqueous H^+^ source (H_2_O + h_VB_^+^ = [O] + 2H^+^).^3^ Thus, proton-coupled multi-electron steps for CO_2_ reductions are generally more favorable than single electron reductions as thermodynamically more stable molecules are produced and the large reorganization energy between the linear molecule and bent radical anion is therefore lowered.^4^ Notably, the choice of innocuous solvent of water is of great interest because it can serve as a simple model for green synthetic methods.

In one approach, the photocatalytic reduction of CO_2_ with H_2_O as a reductant was therefore carried out in aqueous suspension. Halmann *et al.* first showed CO_2_ photoreduction to formic acid on *p*-GaP as photocathode in aqueous media in 1978.^5^ In another approach, the strategy of hybrid photosensitizer with metal complexes as molecular co-catalysts to construct efficient catalytic system was demonstrated.^6^ In particular, a typical photochemical reductive system, initially presented by Lehn *et al.*, contains a catalyst combination of [Ru(bpy)_3_]Cl_2_ (bpy = 2,2′-bipyridine) and CoCl_2_ as a light sensitizer and an electron mediator, respectively.^7^ This reaction system has been widely investigated as a model reaction by follow-up studies.^8^ The protons involved in these reduction systems may originate from H_2_O. Another study has shown that the proportion of H_2_O for an optimal efficiency generation of (CO + H_2_) can be fixed at about 20%.^9^ In these studies, acetonitrile (MeCN) and triethanolamine (TEOA) were regularly used as a solvent and sacrificial agent.

Recently, we found that the reaction medium (MeCN/TEOA) with an appropriate ratio of water divides into two phases during reaction, which is likely in response to the efficiency of photocatalytic CO_2_ reduction. However, a better understanding of the multiple roles of water during CO_2_ conversion is still highly required, involving charge transfer intermediate products and interfacial effect as well as the relationship between physicochemical property and photocatalytic activity. Herein, a systematic investigation was conducted to uncover the mechanism of promotional effect from water on the photocatalytic activity of CO_2_ reduction. This typical reaction was performed using a catalyst combination of CdS as a photocatalyst and [Co(bpy)_3_]Cl_2_ as an electron mediator using TEOA as an electron donor. Moreover, in extended experiments it was shown that a wide variety of organic solvents were examined in such a system, indicating that a major function of the new system is simply for charge separation and mass transfer.

As illustrated in Fig. 1, the yield of production of (CO/H_2_) is closely related to the concentration of water in MeCN solution. When the photoredcution of CO_2_ is conducted in MeCN without water, the generation of CO is moderate (16 μmol). This value is close to that reported previously, which is obtained in a hybrid CO_2_ photoreduction system by mixing CdS nanoparticles and CoCl_2_/bpy in an acetonitrile/TEOA solution.^10^ Clearly, the test method in the absence of water is proved reproductive because the mentioned experimental conditions are quite similar to the current system.

**Fig. 1:**
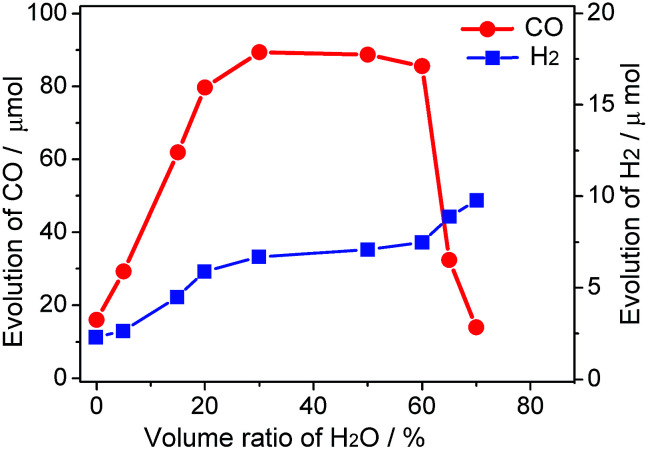
Effect of the water concentration on the evolution of CO/H_2_ in H_2_O–MeCN system.

The role of water in the photocatalytic CO_2_ reduction system is further demonstrated. Addition of water from 0% to 30% results in drastically increasing evolution rates of CO and H_2_; however, for the system containing water beyond 60%, an increase in the amount of H_2_ evolution and a marginal decrease in the amount of CO evolution are observed. The ascending trend before 30% may be explained by the double-role of water as an H^+^ source and a component of the binary system, while the decrease after 60% tenably attributes to latter reason. Moreover, the relevant physical property of the reaction medium including viscosity and conductivity may also be regulated through this procedure. Interestingly, the amount of CO production remains a stable with the proportion of water ranging from 20–60%. As much as 89 μmol CO was produced from H_2_O–MeCN mixture containing 30% water with 2 h visible light irradiation. This is more than 5-fold higher than the yield of CO in MeCN solution without water. Compared to the production of CO, the yield of H_2_ continually improved by the aid of increasing water concentration, reflecting the role of water as a proton source. The relationship of different catalytic performances for CO and H_2_ production along with varying proportion of water is clearly revealed by their selectivity (Fig. S1†). Moreover, there are trace amounts of other products, such as methane, formic acid and methanol, which can be formed in these activity tests.

Next, the photos for binary system are presented in Fig. 2. At the beginning, CdS disperses in all H_2_O–MeCN solutions and appears as a yellow suspension (Fig. 2a–e). Moreover, Co-complex is also dissolved in the continuous system (Fig. S2†).^11^ The reaction medium with the appropriate amount of water was divided into two phases after 20 min light irradiation (Fig. 2a–d). The line between the two different colors was assigned as the interphase boundary of the two phases. Different volume fractions of the subnatant (denoted as “aqueous phase”) is greatly dependent on the amount of water added. This phenomenon is greatly contrary to our conventional concepts that MeCN and TEOA are completely dissolved in water in normal conditions. To further reveal the ingredient of each phase, tetrachloromethane (TCM) was employed to extract water. As shown in Fig. S3,† water was employed as another continuous phase, and its solubility with MeCN is greatly decreased. The study for more details of separation mechanism is in progress.

**Fig. 2:**
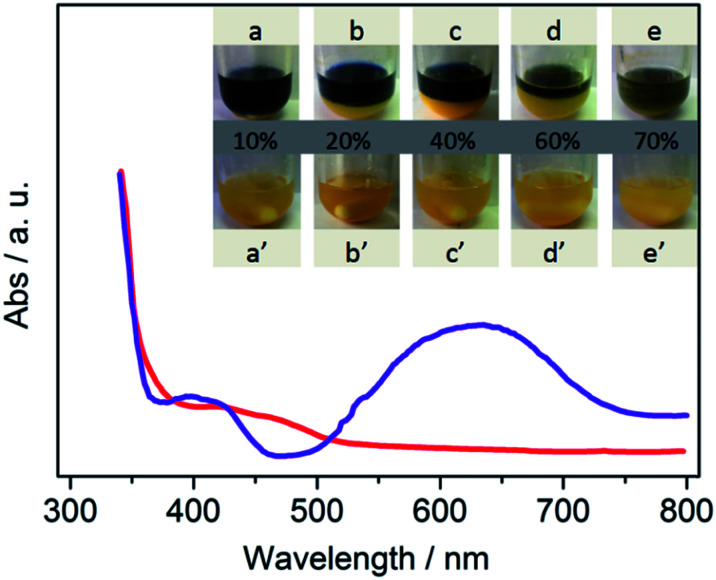
UV-vis absorption spectra of supernatant (blue) and subnatant (red) in H_2_O (40%)–MeCN system after 2 h irradiation. Inserts are the photos of the solution with different water content before (a′–e′) and after irradiation (a–e).

To reveal the differences between them, *in situ* UV-vis spectra of supernatant and subnatant were recorded. The blue color originated from intermediates of Co(i), which has been verified both in organic and aqueous solution in previous studies.^10^ This spectra provides an evidence that the Co^I^ species is mostly concentrated in supernatant (denoted as “organic phase”), which is also confirmed in TCM–H_2_O system (Fig. S4†). Notably, the place where electron generated is successfully separated from the reaction sites, which is greatly prohibited the charge carrier recombination and improved the efficiency of photocatalytic CO_2_ reduction process.

Clearly, the procedure for binary system formation is in good accordance with CO evolution rate. On increasing the amount of water, interfacial liquid–liquid area significantly increased through vigorous stirring. We speculate that the major promotion effect of water may involve phase transfer catalysis (PTC) mechanism.^12^ The binary system was destroyed when the volume ratio of water reached 70% (Fig. 2e), because a small quantity of organic phase can be dissolved in bulk water. Then, the catalytic performance is similar to that in water, while a low CO_2_ conversion can be obtained in aqueous solution. These results indicate that an appropriate organic–aqueous composition provides a platform for the generation of PTC for highly efficient CO_2_ reduction.

The product amounts as a function of reaction time were studied, and the concentration of water was fixed at 30% in this test. As shown in Fig. 3 and S5,† the relationship between the amount of CO/H_2_ produced and the reaction time is non-linear. A different catalytic performance was observed between reaction media in the absence and presence of water. Relatively lower production efficiency in the reaction system without the addition of water was observed in comparison with that in system containing water because organic medium single phase was also unfavorable for charge separation or/and stability of active intermediate. After 2 h illumination, the total production amounts of CO and H_2_ approached a maximum value in H_2_O–MeCN and MeCN system. This is consistent with the damage of active Co^I^ species after several turnovers of the photochemical reactions (Fig. S2(b and c) and S4†). With respect to using water as solvent, trace amount of CO (1.6 μmol) was obtained even after 4 h irradiation.

**Fig. 3:**
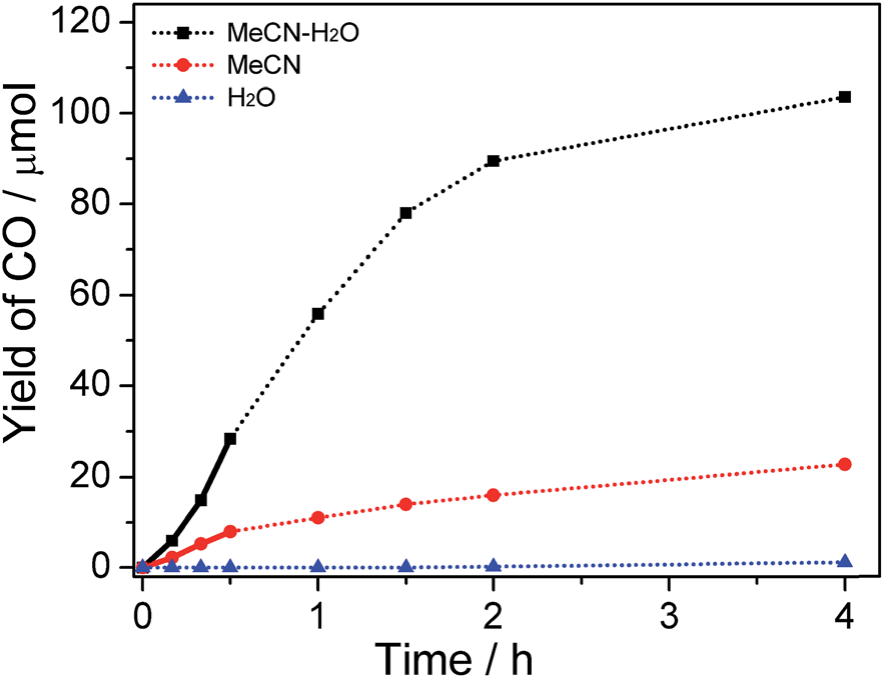
The amount of CO produced as a function of reaction time under visible light illumination.

It should be noted that, compared with the reaction in MeCN, there is an inductive phase when CO_2_ reduction occurs in H_2_O–MeCN mixture (bold line in Fig. 3). At this stage, the generation rate of CO was relatively low. This again proves the presence of binary system that formed from MeCN and H_2_O mixture. This is because the process for binary system construction was required before achievement of highly efficient charge transfer and realization of ultimate CO_2_ conversion. The evolution of H_2_ is also nonlinear and similar in all types of systems (Fig. S5†), indicating that the binary system only works on the reactivity of CO_2_ conversion.

The water-promoted CO_2_ reduction system was then employed in other types of reaction media, to determine the generality of the promotional effect of the binary system and also to search for a favorable water-coupler as reaction medium for efficient CO_2_ photofixation. As shown in Fig. 4 and S6,† upon adding water, all binary systems displayed enhanced photocatalytic reactivity towards CO_2_ and H_2_O reduction. The different reactive performance is related to the intrinsic nature of organic molecule involving polarity, solubility for CO_2_, and coordination ability.^13^ Results revealed that addition of 30% H_2_O in MeCN can significantly increase the CO evolution rate (CER) from 8 to 44.5 μmol h^−1^, and the H_2_ evolution rate (HER) from 3.4 to 6.7 μmol h^−1^. Moreover, an analogical photoreduction performance can be observed in DMF–H_2_O system although a clear interface does not show up in current experiment. The most appropriate proportion of water was fixed at 50% in H_2_O–DMF system with the production rates of 36 μmol h^−1^ and 4.2 μmol h^−1^ for CO and H_2_, respectively.

**Fig. 4:**
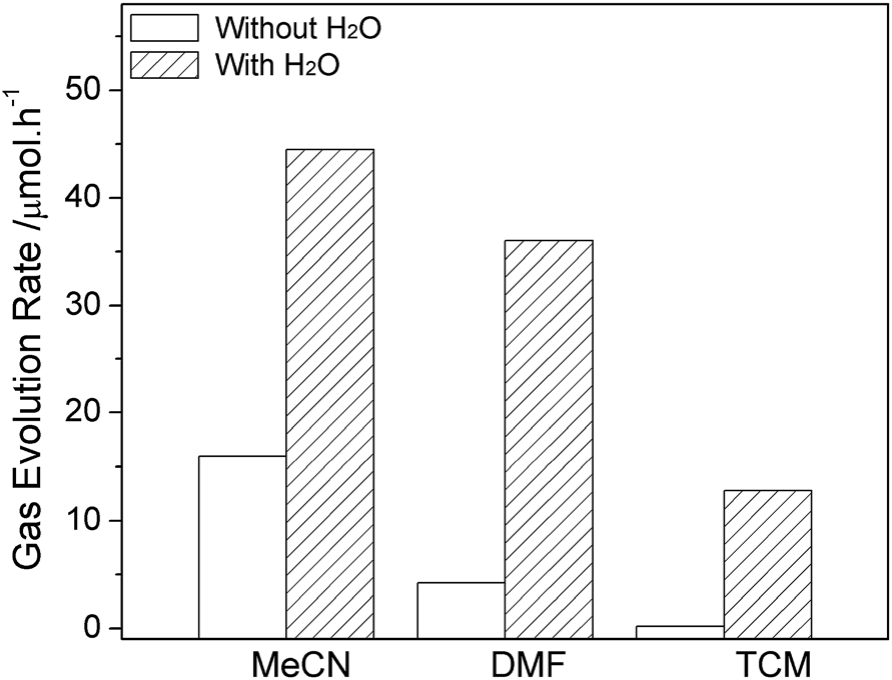
The promotional effect of H_2_O on CO evolution in different solvents (the systems contain optimal amount of water at 30% in MeCN, 50% in DMF and 40% in TCM commixture respectively).

Considering that MeCN and DMF are both soluble in water in normal conditions, an original two phase liquid–liquid system was therefore designed for this test. Instead of former solvents, nonpolar tetrachloromethane (TCM), which is immiscible in water and able to form a binary system prior to reaction, was involved. The CER and HER reach 12.8 μmol h^−1^ and 2.1 μmol h^−1^ in the TCM system consisting of 40% water. There was no gas phase production detected in TCM without H_2_O upon 2 h light irradiation.

Based on previous studies, we proposed the possible process of CO_2_ conversion mediated by cobalt complex in the binary phase system.^7,10^ As shown in Fig. 5, the entire process was divided into six steps. (1) Starting from Co^II^L, the reduction reaction in the metal center occurs after grasping a photogenerated electron by coordinating ligand (bpy) by LMCT process. The generation of Co^I^ has been certified by UV-vis spectra (Fig. 1) and obtained by chemical reduction of [Co(bpy)_3_]^2+^ with Na–Hg.^10^ The functional group of pyridine has been reported as a strong electron acceptor in many types of reactions. (2) Then, a nucleophilic solvent molecule is released from reduced Co centers because the combination strength of these centers is weakened after obtaining one electron. Thus, CO_2_ coordinates with Co^I^L through electrophilic attack. (3) Combined CO_2_ molecular turns into radical anion (CO_2_^−^) by obtaining an electron through MLCT. (4) The subsequent protonations of Co^III^–COO^−^ forms Co^III^–COOH complex. (5) Subsequently, H_2_O and CO successively dissociated from metal center with the aid of surrounding solvent molecules.^14^ (6) Finally, the regeneration of S–Co(i)L_*n*_ occurs through reduction of S–Co(iii)L_*n*_, which initiates the next reaction cycle. The complex Co(bpy)_3_^2+^ has been used as a catalyst for H_2_ generation, and records of the studies are available.^15^ In their modes of reaction, the formation of Co^I^ and Co^III^–H intermediates was proposed to be the key steps for H_2_ generation.

**Fig. 5:**
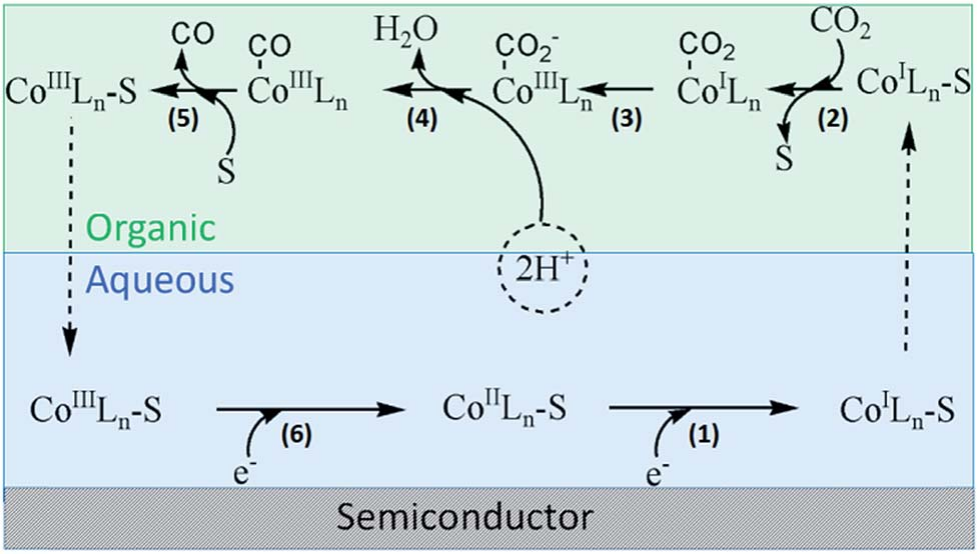
Possible mechanism of photocatalytic CO_2_ reduction in the binary phase system (S: organic solvent molecular, L: ligand of bipyridine).

We evaluated the promotional effect originated from binary phase in the water containing solution. CdS was dispersed in water and floated over CCl_4_ before reaction. However, more sufficient H_2_O (40%) may drag the CdS particles into the bottom of the reactor by gravity after vigorous stirring. Similar phenomenon is also observed in H_2_O–MeCN system under a certain period of irradiation. A blurred interface in H_2_O–DMF system is formed, which may be attributed to the favorable solubility of inorganic salt in DMF. Thus, we can easily accept that the initial step of light harvesting and electron generation occurs in aqueous phase. The step (2) and step (3) may occur at the organic–aqueous interface because metal centers successively obtain CO_2_ and H^+^, while they tend to distribute in organic phase and aqueous phase respectively. In these steps, proton coupled chemical reactions are possible at much less negative potentials than that of one-electron reduction depending on the solvent system.^4^ The mechanism has been proposed such that the selectivity of the formation of CO and/or HCOOH is determined by the adsorption strength of CO_2_^−^ relative to the metal substrate.^16^ In our test, great amount of CO has been produced, while trace amount of HCOOH can be detected. Thus we speculate that the decomposition of Co^III^–COOH complex majorly occurs in organic phase, which possesses relative lower polarity. Another supportive opinion is that the removal of CO from metal center occurs readily with the aid of nucleophilic attack from organic molecules. Thus, the different photocatalytic performances in various solvents may be due to different coordination ability.

Clearly, the construction of a binary liquid–liquid system greatly promotes the photoreduction process of CO_2_. A dynamically feasible scheme for efficient charge transfer and the generation of stable active species was achieved during this reaction. As shown in Fig. 5, cobalt compounds play a vital role of phase transfer catalyst. Moreover, the introduction of cobalt co-catalyst can not only facilitate the charge separation, but also lower the activation energy or overpotential for the reaction. Based on the above analysis, we tried to demonstrate the reduction of CO_2_*via* PTC process.

## Conclusions

In summary, we presented a binary liquid system in the typical photocatalytic CO_2_ reduction reaction by the addition of water. The generation of CO reaches 89 μmol in MeCN solution in 30% water, which is much higher than that in MeCN (16 μmol) or H_2_O (1.6 μmol) as reaction medium. This novel system exhibits high photocatalytic reactivity towards CO_2_-to-CO conversion due to the addition of water as another phase, thus promoting charge-carrier separation, transfer kinetics, and interface interaction. Of particular interest is that the excellent photocatalytic activity in the binary liquid phase can be developed as a universal photocatalytic CO_2_ reduction system. The new findings clearly demonstrate that the integration of water with various organic solvents is a feasible process for the design and development of efficient photochemical systems for CO_2_ conversion.

## Conflicts of interest

There are no conflicts to declare.

## Supplementary Material

RA-008-C7RA12801K-s001
