# Supplementary material for: High-efficiency photocatalytic CO2 reduction in organic–aqueous system: a new insight into the role of water
Source: RSC Adv. 2018 Jan 19;8(7):3798–802. doi: 10.1039/c7ra12801k (PMC9077867; doi:10.1039/c7ra12801k)
Supplement: RA-008-C7RA12801K-s001 [file RA-008-C7RA12801K-s001.pdf]

Electronic Supplementary Material (ESI) for RSC Advances.
This journal is © The Royal Society of Chemistry 2018

Supporting Information

High-efficiency photocatalytic CO_2 reduction in organic-aqueous system: a new insight into the role of water

Jinliang Lin[a] *, Rongying Liao[a] and Junli Xu[b]

[a] Department of Chemical and Engineering, Zunyi Normal College, 563000, Zunyi, P.R. China.

[b] Research Institute of Photocatalysis, State Key Laboratory of Photocatalysis, Fuzhou University, Fuzhou 350002, P.R. China.

Email:jinliang_lin@163.com

Chemicals

2,2`-bipyridine(bpy, Alfa), cobalt chloride hexahydrate ($CoCl_2{\cdot}6H_2O$, Sigma), Cadmium sulfide (CdS, Sigma, 99.9 %) were used as received. All the other chemicals, including N, N-dimethylformamide (DMF), tetrachloromethane (TCM), acetonitrile (MeCN), triethanolamine (TEOA) are of reagent grade, were purchased from China Sinopharm Chemical reagent Co. Ltd and without further purification. The used water is ultrapure with resistivity of *ca.*18 MΩ·cm.

Characterization

Absorption spectra were obtained on an UV-Vis spectrophotometer (Varian Cary 50 Conc). The test samples were reaction mediums before reaction, supernatant and subnatant of reaction medium after reaction, respectively. Detection of CoI transition was conducted in a sealed container adaptable for record equipment, and exposed light irradiation for 20 min before test. Photos of Co^I complex was took in a glass reactor, and exposed light irradiation for 20 min or 120 min.

Photocatalysis test

All experiments were performed in a Schlenk flask (80 ml) under an atmosphere pressure of CO_2 (1 atm). In the Schlenk flask, CdS (50mg), bpy(15mg), $CoCl_2{\cdot}6H_2O$ (5 μmol) were added in the mixture of water-solvent (total volume: 5 ml) and triethanolamine (TEOA, 1 ml). TEOA was used as the sacrificial agent. The system was subjected to vacuum degassing and backfilling with pure CO_2 gas. This process was repeated three times and after the last cycle the flask was back filled with CO_2. Then the system was irradiated with six counter non-focus 50 W white LED light source under vigorous stirring at 20 ^{o}C controlled by a water-cooling system. The produced gases (CO, H_2) were detected by a gas chromatography (Agilent 7890, Agilent Technologies) equipped with a packed column (TDX-1 mesh 42/10). Ar was used as the carrier gas.

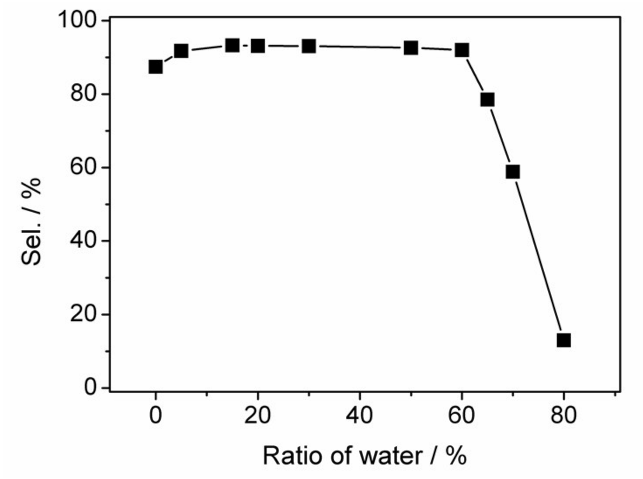

Fig. S1 Selectivity of CO in system with various water ratio.

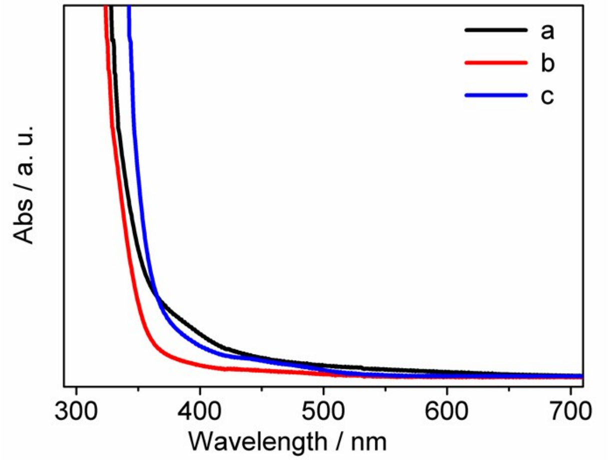

Fig. S2 UV-Vis absorption spectra of different samples: (a) Before reaction, (b) supernatant of solution after reaction, and (c) subnatant of solution after reaction.

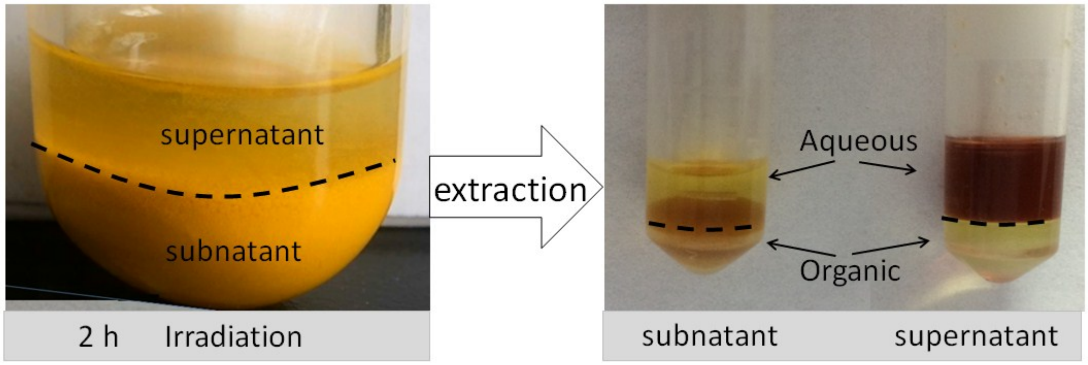

Fig. S3 Illustration of aqueous/organic components in supernatant and subnatant of solution after 2h irradiation (extracted by tetrachloromethane).

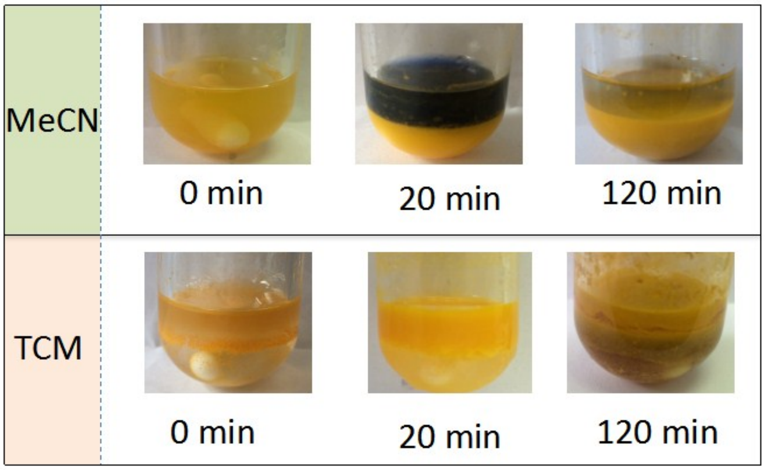

Fig. S4 The reaction medium irradiated by visible light with different duration.

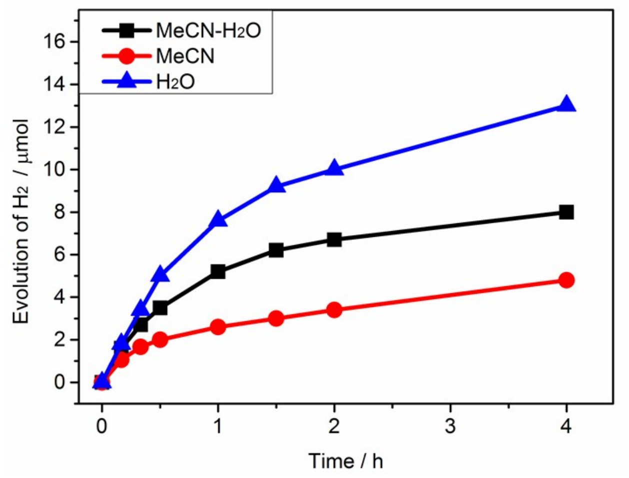

Fig. S5 The evolution of H_2 in different reaction medium as a function of reaction time.

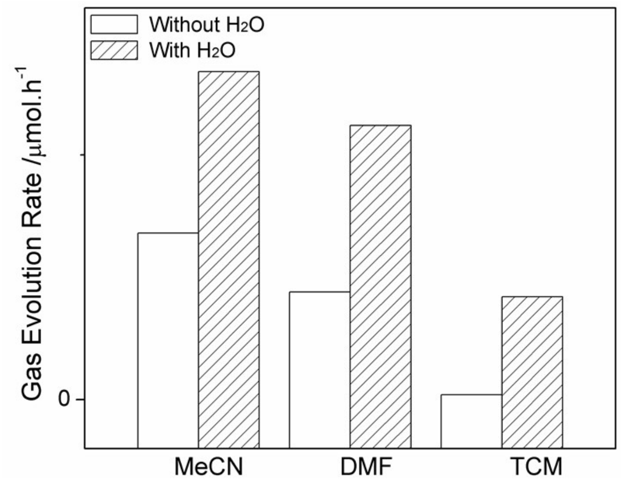

Fig. S6 The effects of water on HER in various solvents. (The systems contain optimal amount of water at 30% in MeCN, 50% in DMF, and 40% in TCM solution respectively.)
